# Endothelial Nox5 Expression Modulates Glucose Uptake and Lipid Accumulation in Mice Fed a High-Fat Diet and 3T3-L1 Adipocytes Treated with Glucose and Palmitic Acid

**DOI:** 10.3390/ijms22052729

**Published:** 2021-03-08

**Authors:** Jorge G. García, Eduardo Ansorena, Fermín I. Milagro, Guillermo Zalba, Carlos de Miguel

**Affiliations:** 1 Department of Biochemistry and Genetics, University of Navarra, 31008 Pamplona, Spain; jgarcia.51@alumni.unav.es (J.G.G.); eansorena@unav.es (E.A.); gzalba@unav.es (G.Z.); 2 Navarra Institute for Health Research (IdiSNA), 31008 Pamplona, Spain; fmilagro@unav.es; 3 Center for Nutrition Research, Department of Nutrition, Food Science, and Physiology, University of Navarra, 31008 Pamplona, Spain; 4 Centro de Investigación Biomédica en Red Fisiopatología de la Obesidad y Nutrición (CIBERobm), Instituto de Salud Carlos III, 28029 Madrid, Spain

**Keywords:** obesity, NADPH oxidase 5, lipid homeostasis, glucose uptake, Caveolin 1, Glut4

## Abstract

Obesity is a global health issue associated with insulin resistance and altered lipid homeostasis. It has been described that reactive oxygen species (ROS) derived from nicotinamide adenine dinucleotide phosphate (NADPH) oxidase (NOX) activity are involved in the development of these pathologies. The present study describes the role of endothelial NOX5 expression over adipose tissue by using two experimental systems: NOX5 conditional knock-in mice fed with a high-fat diet and 3T3-L1 adipocytes cultured with conditioned media of NOX5-expressing endothelial cells previously treated with glucose and palmitic acid. Animals expressing NOX5 presented lower body weight gain and less mesenteric and epididymal adipose mass compared to control mice fed with the same diet. NOX5-expressing mice also showed significantly lower glycaemia and improved insulin-induced glucose uptake. In addition, *Glut4* and Caveolin 1 (*Cav1*) expression were significantly increased in the adipose tissue of these animals. Likewise, 3T3-L1 adipocytes treated with conditioned media from NOX5-expressing endothelial cells, incubated with high glucose and palmitic acid, presented a reduction in lipid accumulation and an increase in glucose uptake. Moreover, a significant increase in the expression of *Glut4* and *Cav1* was also detected in these cells. Taken together, all these data support that, in response to a highly caloric diet, NOX5 endothelial activity may regulate glucose sensitivity and lipid homeostasis in the adipose tissue.

## 1. Introduction

Obesity is considered a global health issue characterised by an increase in body weight that results in an excessive fat accumulation, disrupting adipose tissue homeostasis [1]. Obesity could lead to metabolic syndrome development, insulin resistance, and type 2 diabetes, which in turn might develop into liver and cardiovascular disorders. [2]. Insulin resistance is defined as a condition produced by the low or inexistent response to high levels of insulin in the organism. In normal conditions, insulin binds to insulin receptors (IR), promoting its activation and its auto-phosphorylation, triggering the translocation of glucose transporter protein (GLUT4) to the cell membrane [3]. Effective insulin signalling in adipocytes may be primarily dependent on the localization of insulin responsive elements (IR and GLUT4) to *caveolae*, small invaginations of the plasma membrane that are especially abundant in this type of cell. *Caveolae*, considered signalling platforms, facilitate a rapid activation of IR improving signal transduction [4]. The main structural and functional components of *caveolae* are caveolins, cholesterol-dependent integral membrane proteins [5]. It has been pointed out that caveolin 1 (CAV1) presents a direct functional interaction with the β-subunit of the IR, enhancing insulin signal transmission [4]. Moreover, CAV1 emerges also as pivotal for the integration of GLUT4 within the plasma membrane [6]. On the other hand, CAV1 has also been involved in adipose tissue homeostasis regulating lipid accumulation [7,8].

One of the mechanisms underlying the relationships among obesity, metabolic syndrome, and type 2 diabetes is the increased production of reactive oxygen species (ROS) [9,10]. These increased levels of ROS could act as drivers of redox signalling or even induce oxidative stress when exceeding the balance between their production and degradation [11]. In eukaryotic cells, ROS are commonly generated as products of normal aerobic metabolism and participate in cellular signalling. One of the main producers of ROS in the organism is the family of nicotinamide adenine dinucleotide phosphate (NADPH) oxidases [12]. The NADPH oxidase (NOX) family is formed by seven members: homologous *NOX1*–*5* and dual oxidases *DUOX1*-*2*. These transmembrane proteins are considered professional oxidases as their primary function is ROS production [13]. 

Within this family, nicotinamide adenine dinucleotide phosphate (NADPH) oxidase 5 (NOX5) is the most recently discovered member [14,15]. This isoform presents a characteristic structural difference consisting in the presence of four EF-hand motifs in the N-terminal part of the protein, which makes NOX5 activity calcium-dependent. Furthermore, it does not need the binding of p22phox subunit for being active [14]. There are six possible splicing variants described, but only the isoforms 1 (α) and 2 (β) are catalytically active [16]. Some of its biological functions or its involvement in pathophysiological processes remain unclear due to its evolutionary loss in the genome of common laboratory rodent models. In humans, the functional isoforms α and β are expressed in the vasculature among other tissues, with β isoform being the predominant in the endothelial cells of the intima [16,17].

NOX enzymes have been implicated in the pathology of metabolic disorders [18]. Nutrients such as glucose and fatty acids play a regulatory role in the expression and activity of some NOX family members, and some of these proteins contribute to obesity-associated insulin resistance [19]. Additionally, ROS plays a role in adipocyte differentiation [20,21]. Interestingly, some studies have proved that NOX4-derived superoxide enhances glucose uptake as well as adipocyte differentiation [21,22]. A direct interaction between *caveolae* and NOX enzymes has also been shown. A recent study has revealed that NOX5 is present in *caveolae* from vascular smooth muscle cells [23]. In this regard, it has been described that CAV1 can bind to NOX5 promoting a decrease of its activity [24]. There is a close relationship between metabolic tissues and the vasculature, and an increasing amount of evidence illustrates that there are intense cross-signalling between them [25]. Understanding the physiological and pathophysiological roles of vascular NOX5 in the development and progression of metabolic diseases is desirable since obesity and type 2 diabetes entail a greater risk of cardiovascular mortality [18]. In this context, the aim of this study was to determine whether endothelial NOX5 expression in mice fed with a high-fat diet (HFD) could have an effect in insulin signalling and the homeostasis of adipose tissue. For that purpose, a novel humanised NOX5 conditional knock-in mice, developed by our group [26], was used. The principal finding of this work shows that endothelial NOX5 expression modulated glucose uptake and lipid accumulation in the adipose tissue of mice fed with a high-fat diet, accompanied with a significant increase in the expression of GLUT4 and CAV1. This effect was confirmed in cultured 3T3-L1 adipocytes incubated with conditioned media released from NOX5-expressing endothelial cells exposed to high levels of glucose and palmitic acid.

## 2. Results

### 2.1. Endothelial NOX5 Expression Reduced Body Weight Gain and Adipose Tissue Weight in Mice Fed a High-Fat Diet

**Figure 1:**
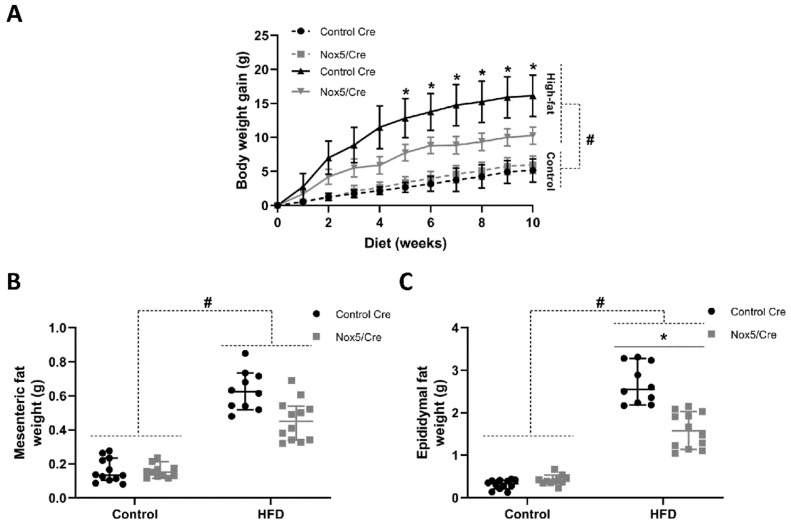
Endothelial NOX5expression reduced total body weight gain and the amounts of mesenteric and epididymal adipose tissues in mice fed a HFD for 10 weeks. (**A**) Body weight gain. (**B**) Mesenteric fat weight. (**C**) Epididymal fat weight. Control diet: control Cre (*n* = 12), Nox5/Cre (*n* = 11); high-fat diet: control Cre (*n* = 10), Nox5/Cre (*n* = 12). Values are expressed as median with confidence interval. # *p* < 0.05: diet differences (dotted lines); * *p* < 0.05: genotype differences (solid lines). Statistical tests used: Aligned Rank ANOVA.

Control CRE recombinase (CRE) or endothelial NOX5-expressing mice were fed either with a control or a high-fat diet (HFD) for 10 weeks. Body weight gain was determined each week and organ tissue weights were measured at the time of sacrifice. Body weight gain experienced a continuous increase over the 10 weeks of treatment in both groups, becoming significantly higher after the second week of treatment in those animals fed with the HFD. More importantly, endothelial NOX5-expressing mice fed with the HFD presented lower body weight gain than their control CRE counterparts. This decrease, caused by the difference in genotype, became significant since the fifth week of diet (Figure 1A). Accordingly, weights of mesenteric and epididymal fat tissues from mice fed with the HFD were significantly increased after 10 weeks of treatment compared to mice fed a control diet. Noteworthy, endothelial NOX5 expression tended to reduce the weight of mesenteric and epididymal fat in mice fed with HFD compared to their control CRE counterparts. This decrease was significant for epididymal fat (Figure 1B,C).

### 2.2. Endothelial NOX5 Expression in Mice Fed a High-Fat Diet Enhanced Glucose Sensitivity

**Figure 2:**
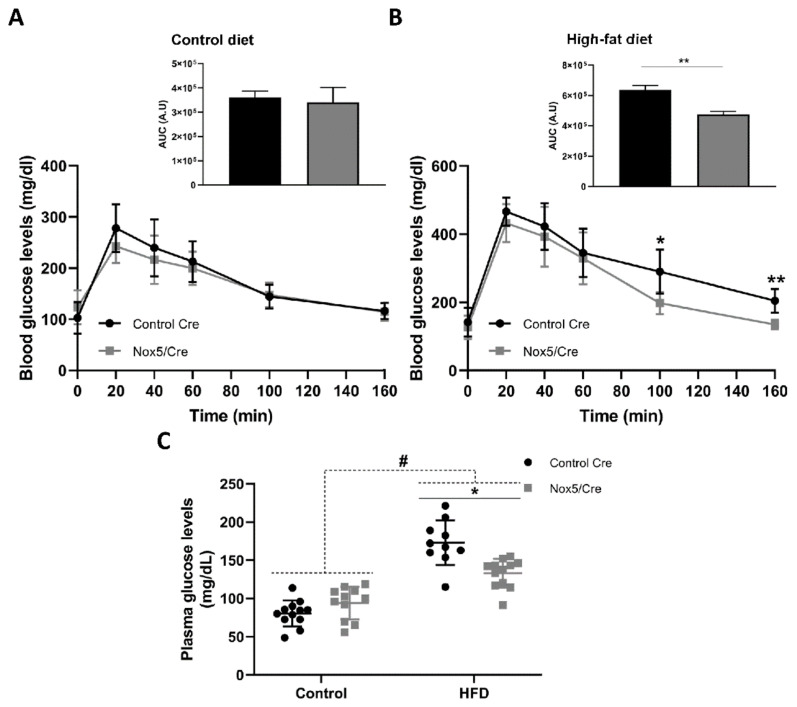
Endothelial NOX5 expression improved glucose uptake and reduced glycaemia in mice fed a HFD. (**A**) Intraperitoneal glucose tolerance test (IPGTT) performed in animals fed a control diet for 9 weeks. (**B**) IPGTT performed in animals fed a HFD for 9 weeks. (**C**) Plasma glucose levels after 10 weeks. Control diet: control Cre (*n* = 12), Nox5/Cre (*n* = 11); high-fat diet: control Cre (*n* = 10), Nox5/Cre (*n* = 12). Values are expressed as mean ± standard error of the mean (SEM). # *p* < 0.05: diet differences (dotted lines); * *p* < 0.05, ** *p* < 0.01: genotype differences (solid lines). Statistical tests used: *t*-test for (**A**,**B**), and area under the curve (AUC); two-way ANOVA for (**C**). A.U: arbitrary units.

An intraperitoneal glucose tolerance test was performed measuring blood glucose levels in mice fed either with a control or a HFD for 9 weeks, 1 week before sacrifice. As expected, diet effect provoked that mice fed with the HFD presented higher blood glucose levels throughout the analysed period, compared to mice fed a control diet (Figure 2A,B). In mice fed a control diet, no differences were patent due to genotype. Interestingly, within the group fed with the HFD, mice expressing endothelial NOX5 presented lower blood glucose levels. This difference became significant 100 and 160 min after the intraperitoneal administration of glucose, indicating that the difference in genotype modulated glucose uptake. Thus, NOX5 expression seemed to improve glucose sensitivity in these conditions (Figure 2B).

Biochemical analysis revealed that plasma glucose levels were also significantly increased in mice fed the HFD for 10 weeks (Figure 2C). Differences in genotype provoked that among animals fed with the HFD, mice expressing endothelial NOX5 experienced a significant reduction in glycaemia (Figure 2C). Other biochemical parameters determined from plasma samples showed that mice expressing endothelial NOX5 and fed with the HFD tended to present lower levels of cholesterol, high-density lipoprotein (HDL), triglycerides (TAG), and alanine transaminase (ALT) than control CRE mice (Table S1). Additionally, ROS production was indirectly measured by detecting albumin nitrotyrosine levels in mesenteric fat (Figure S1). Nitrotyrosine levels of this protein were significantly increased in mice fed the HFD for 10 weeks. Moreover, differences in genotype caused that among animals fed the HFD, mice expressing endothelial NOX5 experienced a significant increase in albumin nitrotyrosine levels, reflecting an increase in ROS production.

### 2.3. Endothelial Expression of NOX5 in Mice Fed a High-Fat Diet Increased the Expression of Genes Related to Glucose Uptake Pathway in Adipose Tissue

**Figure 3:**
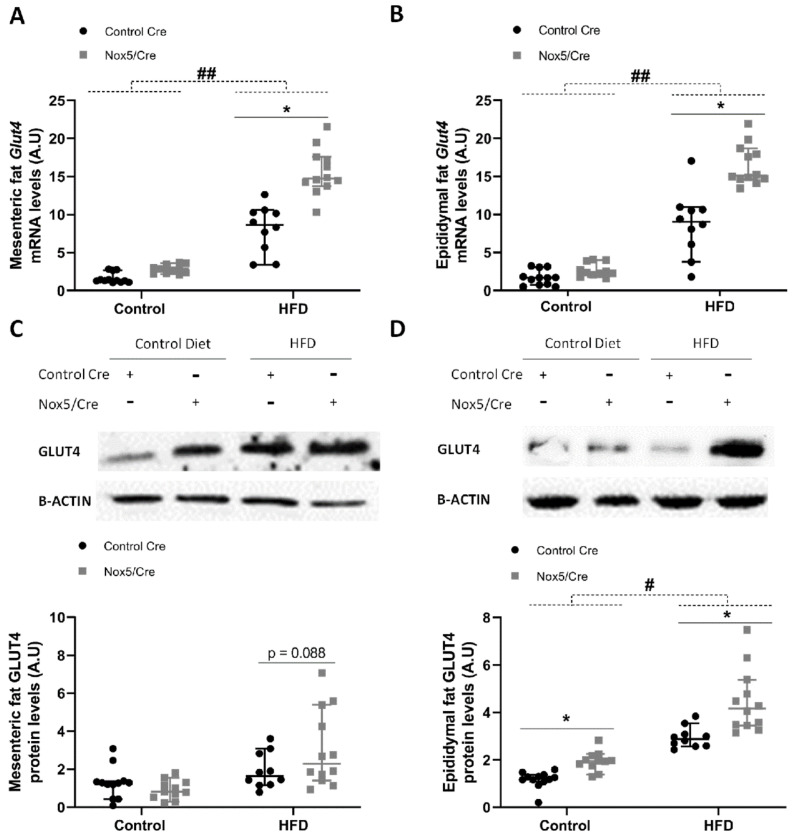
Endothelial NOX5 expression increased Glut4 levels in mesenteric and epididymal fat in mice fed a HFD for 10 weeks. (**A**,**B**) Glut4 mRNA levels in (**A**) mesenteric fat and (**B**) epididymal fat. (**C**,**D**) Representative Western blot and protein levels of GLUT4 in total lysates of (**C**) mesenteric fat and (**D**) epididymal fat. Control diet: control Cre (*n* = 12), Nox5/Cre (*n* = 11); high-fat diet: control Cre (*n* = 10), Nox5/Cre (*n* = 12). Values are expressed as median with confidence interval. mRNA levels are relative to *Gapdh.* Protein levels are relative to B-ACTIN. # *p* < 0.05, ## *p* < 0.01: diet differences (dotted lines); * *p* < 0.05: genotype differences (solid lines). Statistical test used: Aligned Rank ANOVA.

The expression of different genes associated with glucose sensitivity was evaluated in the adipose tissue of animals subjected to control or to a HFD for 10 weeks. Glut4 expression was evaluated in samples obtained from mesenteric and epididymal fat. The HFD group showed that mRNA levels of Glut4 were significantly higher in both tissues. In addition, endothelial NOX5 expression in mice fed the HFD induced a significant increase of Glut4 mRNA levels in both tissues, showing relevant differences due to genotype (Figure 3A,B). This NOX5 genotype effect on Glut4 expression was confirmed at protein levels in epididymal fat, where it became clear even with the control diet (Figure 3D). On the other hand, in mesenteric fat, only those animals fed a HFD and expressing endothelial NOX5 showed a tendency to increase GLUT4 protein levels (Figure 3C).

**Figure 4:**
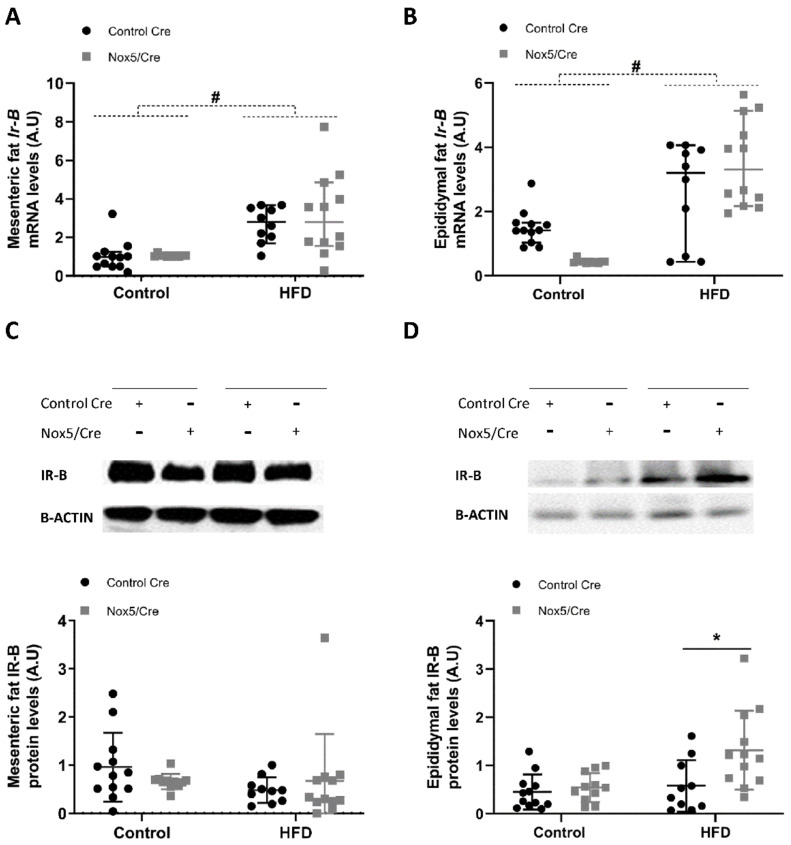
Endothelial NOX5 expression increased Ir-B expression in epididymal but not in mesenteric fat in mice fed a HFD for 10 weeks. (**A**,**B**) Ir-B mRNA levels in (**A**) mesenteric fat and (**B**) epididymal fat. (**C**,**D**) Representative Western blot and protein levels of IR-B in total lysates of (**C**) mesenteric fat and (**D**) epididymal fat. Control diet: control Cre (*n* = 12), Nox5/Cre (*n* = 11); high-fat diet: control Cre (*n* = 10), Nox5/Cre (*n* = 12). Values are expressed as median with confidence interval. mRNA levels are relative to *Gapdh*. Protein levels are relative to B-ACTIN. # *p* < 0.05: diet differences (dotted lines); * *p* < 0.05: genotype differences (solid lines). Statistical test used: Aligned Rank ANOVA.

In the case of Ir-B, the HFD feeding induced increased levels of mRNA in both mesenteric and epididymal fat, whereas NOX5 endothelial expression did not elicited appreciable differences due to the genotype in either of the diet conditions (Figure 4A,B). At protein levels, NOX5 endothelial expression only induced a significant increase of IR-B in epididymal fat (Figure 4C,D).

**Figure 5:**
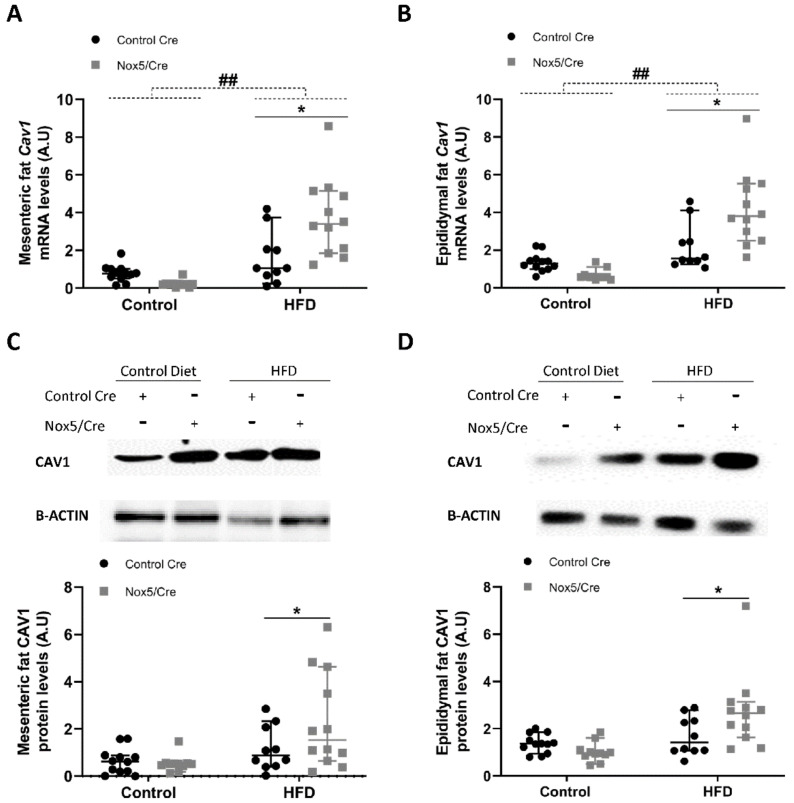
Endothelial NOX5 expression increased Cav1 expression in mesenteric and epididymal fat in mice fed a HFD for 10 weeks. (**A**,**B**) Cav1 mRNA levels in (**A**) mesenteric fat and (**B**) epididymal fat. (**C**,**D**) Representative Western blot and protein levels of CAV1 in total lysates of (**C**) mesenteric fat and (**D**) epididymal fat. Control diet: control Cre (*n* = 12), Nox5/Cre (*n* = 11); high-fat diet: control Cre (*n* = 10), Nox5/Cre (*n* = 12). Values are expressed as median with confidence interval. mRNA levels are relative to *Gapdh*. Protein levels are relative to B-ACTIN. ## *p* < 0.01: diet differences (dotted lines); * *p* < 0.05: genotype differences (solid lines). Statistical test used: Aligned Rank ANOVA.

For Cav1, administration of a HFD induced a significant increase in mRNA levels in both adipose tissues (Figure 5A,B). Additionally, endothelial NOX5 expression had a remarkable effect in Cav1 expression also in both adipose tissues, showing a significant increase, not only of mRNA, but also of protein levels (Figure 5A–D). IR and Cav1 were also analysed in liver samples, where endothelial NOX5 expression in mice fed with the HFD showed a tendency to increase CAV1 protein levels (Figure S2).

Taking all this data into account, we could infer that endothelial expression of NOX5 in mice modulates the expression of some relevant genes related to glucose uptake in the adipose tissue of mice fed with a HFD.

### 2.4. T3 Adipocytes Cultured with Conditioned Media from Endothelial NOX5-Expressing Cells Showed Reduced Lipid Accumulation and Increased Glucose Uptake Together with Enhanced Expression of Genes Related to Glucose Uptake Pathway

An in vitro cell-based model system employing 3T3-L1 adipocytes and NOX5-transfected bEnd.3 endothelial cells was developed in order to confirm the results obtained in the in vivo model with the HFD-fed mice. For that purpose, 3T3-L1 adipocytes were treated for 24 h with conditioned media from NOX5-expressing bEnd.3 endothelial cells, which had been previously incubated with 30 mM glucose and 300 µM palmitic acid for 24 h (Glu + PA conditioned media). Addition of glucose and palmitic acid was performed 24 h after the transfection of the endothelial cells with NOX5 expression vector and was intended to simulate the conditions found in animals fed with a HFD, having high concentration of glucose and fatty acids. NOX5 expression and ROS levels derived from its activation were determined in bEnd.3 cells (Figure S3).

**Figure 6:**
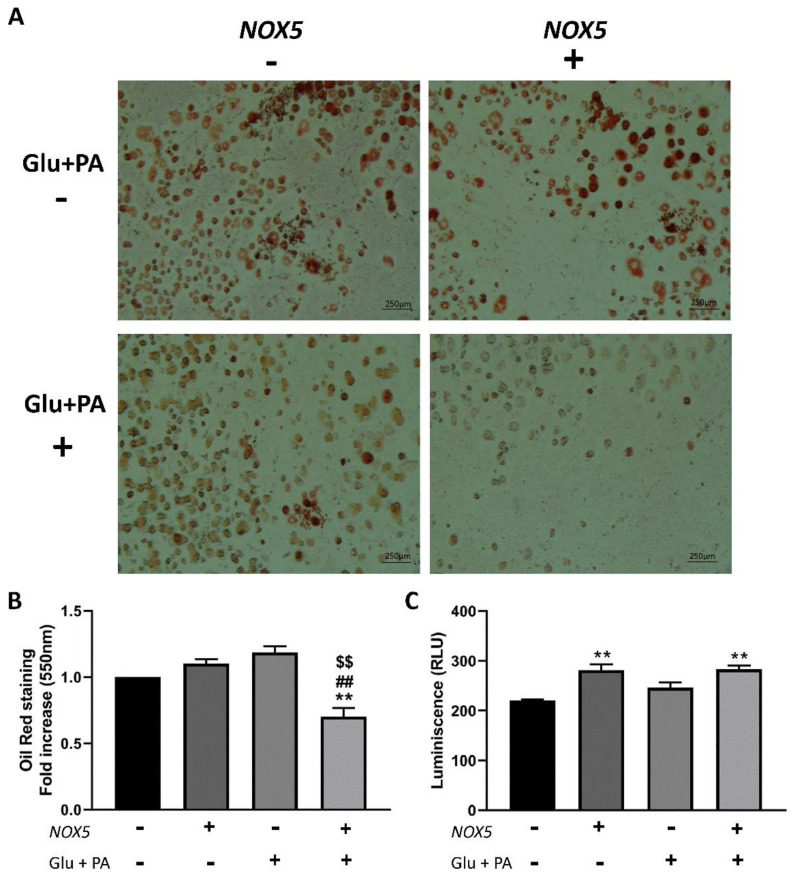
3T3-L1 adipocytes showed a reduction of lipid accumulation when incubated for 24 h with glucose (Glu) + palmitic acid (PA) conditioned media from NOX5-expressing endothelial bEnd.3 cells and an increase of glucose uptake even without the previous incubation with Glu + PA. (**A**) Representative images of Oil Red staining (*n* = 3). (**B**) Oil Red quantification at 550 nm (*n* = 3). (**C**) Glucose uptake quantification of 3T3-L1 adipocytes through luminescence after stimulation with 100 nM insulin. Fold increase is relative to the control group (Nox(−)/Glu + PA(−)). Values are expressed as mean ± SEM. ** *p* < 0.01: differences relative to Nox(−)/Glu + PA(−); ## *p* < 0.01: differences relative to Nox(+)/Glu + PA(−); $$ *p* < 0.01: differences relative to Nox(−)/Glu + PA(+). Statistical test used: ANOVA.

In order to measure the accumulation of neutral triglycerides and lipids in the in vitro system, 3T3-L1 adipocytes were stained with Oil Red after treatment with conditioned media obtained from the endothelial cells. As it can be inferred from Figure 6A,B, only Glu + PA conditioned media derived from NOX5-expressing cells induced a significant reduction of lipid accumulation.

In addition, after 100 nM insulin stimulation, glucose uptake of 3T3-L1 adipocytes treated under the different experimental conditions was analysed. In this case, 3T3-L1 adipocytes treated with conditioned media from NOX5-expressing cells presented a significant increase in glucose uptake independently of the previous incubation of these cells with Glu + PA (Figure 6C).

**Figure 7:**
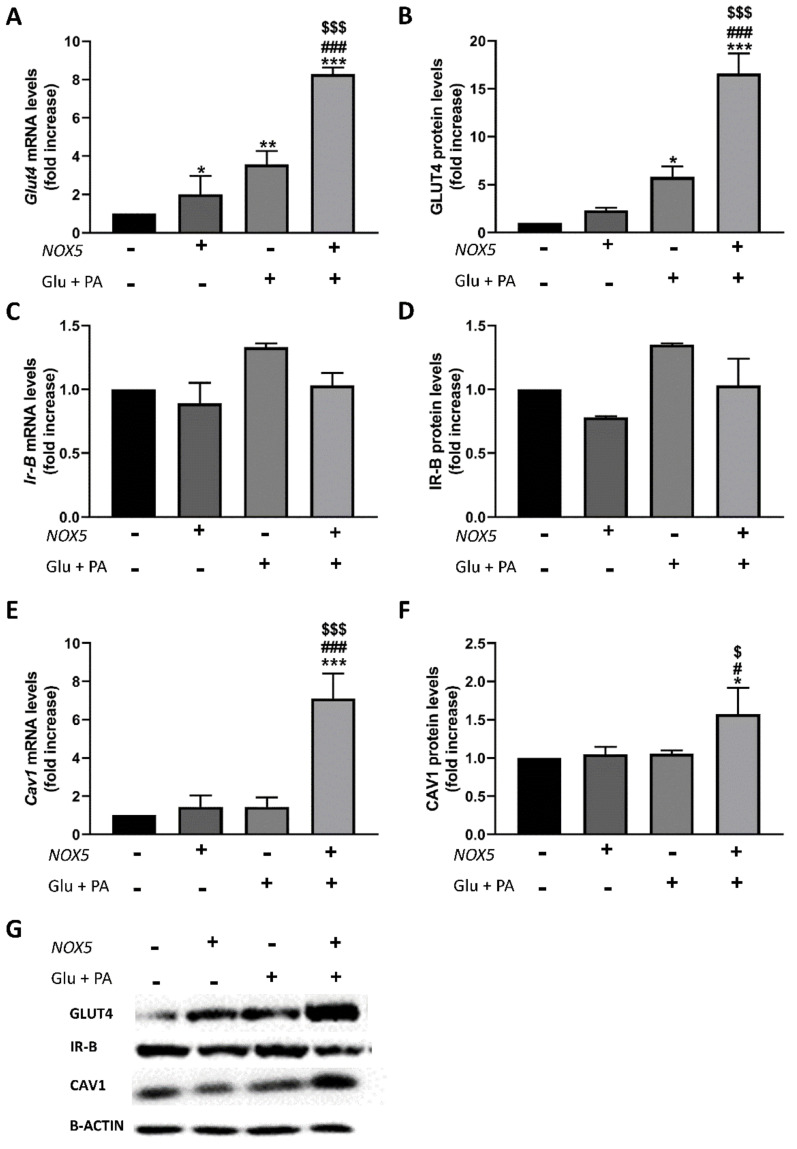
Glut4 and Cav-1 expression was increased in 3T3-L1 adipocytes when incubated for 24 h with Glu + PA conditioned media from NOX5-expressing endothelial bEnd.3 cells. (**A**,**C**,**E**) Glut4, Ir-B, and Cav1 mRNA levels of 3T3-L1 adipocytes (*n* = 3). (**B**,**D**,**F**) GLUT4, IR-B, and CAV1 protein levels of 3T3-L1 adipocytes (*n* = 3). (**G**) Representative images of Western blot for GLUT4, IR-B, and CAV1. mRNA levels are relative to GAPDH; protein levels are relative to β-actin. Fold increase is relative to the control group (Nox(−)/Glu + PA(−)). Values are expressed as mean ± SEM; * *p* < 0.05, ** *p* < 0.01, *** *p* < 0.001: differences relative to Nox(−)/Glu + PA(−); # *p* < 0.05, ### *p* < 0.001: differences relative to Nox(+)/Glu + PA(-); $ *p* < 0.05, $$$ *p* < 0.001: differences relative to Nox(−)/Glu + PA(+). Statistical test used: ANOVA.

Finally, the expression of the relevant genes associated with glucose uptake in adipocytes (Glut4, Ir-B, and Cav1) (Figure 7) was also analysed. In agreement with the previous in vivo mice model results, 3T3-L1 adipocytes treated with Glu + PA conditioned media derived from NOX5-expressing endothelial cells experienced a significant increase in mRNA and protein levels of Glut4 (Figure 7A,B) and Cav1 (Figure 7E,F).

Taken together, these observations indicate that the exposure of endothelial cells expressing NOX5 to high concentrations of glucose and fatty acids (palmitic) prompt the production of molecular signals that cause a reduction in lipid accumulation and an increase in insulin-induced glucose uptake in the neighbouring adipocytes.

## 3. Discussion

Obesity is recognized as one of the leading health problems affecting western countries. One of the circumstances generally associated with obesity is oxidative stress, which is the result of an imbalance between ROS production and degradation [27]. NADPH oxidases (NOXs) are one of the main contributors to ROS homeostasis. Several studies have reported the implication of different NOXs, especially NOX2 and NOX4, in the development of insulin resistance associated with obesity [11,28,29]. However, little is known about the role of NOX5 due to its evolutionary loss in the rodent genome. A recent study shows that pancreatic NOX5 expression in presence of a HFD impairs insulin action [30]. The expression of NOX5 has been frequently associated as a response to an insult or a stress [31] and has been commonly related to a deterioration of the pathology [32]. On the other hand, other studies suggest that NOX5 expression could improve some conditions such as cardiac remodelling or monocytic differentiation into dendritic cells [26,33,34].

In our study, endothelial NOX5 expression seemed to reduce body weight gain and adipose tissue enlargement in mice fed a HFD (Figure 1). Biochemical parameters related to obesity (cholesterol, TAG, glucose) were also reduced in these animals (Table S1, Figure 2). Furthermore, in vitro studies show that 3T3-L1 adipocytes cultured with Glu + PA conditioned media from NOX5-expressing bEnd.3 endothelial cells presented lower lipid accumulation (Figure 6A,B). In accordance with our results, a knockout mice model for NOX4 seemed to facilitate weight gain and obesity development [35]. Although we cannot conclude whether our results are related to molecular signals derived from NOX5 activity, other studies have shown a similar relationship between ROS and lipid homeostasis. ROS derived from mitochondrial activity from the hypothalamus have been reported to control lipid sensing and satiety [36,37]. As control Cre and NOX5/Cre-expressing mice fed with control diet presented no differences, our results suggest that in the presence of a high energy supply, such as feeding with a HFD, the endothelial NOX5 expression might help in body weight control.

In this work, we also found that mice expressing endothelial NOX5, when challenged with a HFD, showed a better response to the IPGTT (Figure 2A,B), presenting a significantly lower blood glucose level 100 min after glucose injection, which reflected an improved glucose uptake response. The in vitro model of 3T3-L1 adipocytes treated with Glu + PA conditioned media from NOX5-expressing bEnd.3 endothelial cells confirmed this result (Figure 6C). These cells showed an increased insulin-stimulated glucose uptake compared to adipocytes treated with Glu + PA conditioned media from non-transfected bEnd.3 endothelial cells. Interestingly, in this case, 3T3-L1 adipocytes treated with conditioned media from NOX5-expressing bEnd.3 endothelial cells, but without previous incubation with Glu + PA, also showed increased insulin stimulated glucose uptake. It seems that in this isolated cell model, NOX5 expression in the endothelial cell was sufficient to ameliorate insulin response in the adipocytes. These results suggest that ROS production in the endothelial cells, derived from NOX5 expression, induces the generation of molecular signals that when reaching the neighbouring adipocytes are able to elicit a response that improves insulin-stimulated glucose uptake in these cells. In vivo, this effect seems to be appreciable only in HFD-fed animals, perhaps as an initial adaptive defence mechanism against the nutrient excess. HFD would increase calcium entrance into the cells [38], allowing NOX5 activation. NOX5-derived ROS could regulate glucose homeostasis, eliciting the production of signals in a less direct way than improved insulin signalling by NOX4-derived superoxide [22].

On the other hand, it is generally accepted that a prolonged situation of over-excess feeding comes down to the development of insulin resistance and type 2 diabetes [39]. Even though it has been reported that oxidative stress plays an important role in this process [20], some studies have proven that basal levels of ROS are essential for insulin sensitivity [40]. For example, a *Gpx1* (glutathione peroxidase 1) knockout mice model produced an increase in ROS levels that resulted in an improved insulin sensitivity through Pi3k/Akt signalling. In another model, mice with an accelerated skeletal muscle senescence showed an increase of mitochondrial ROS levels that resulted in a better sensitivity towards insulin [41,42]. To further analyse the improvement in insulin response as a consequence of NOX5 expression, we measured the expression levels of two initial intermediaries in insulin signal transmission, namely, *Ir-B* and *Cav1*, as well as the final effector, glucose transporter *Glut4*. In accordance with previous results [43], mice fed a HFD showed elevated expression of the three intermediaries in mesenteric and epididymal white adipose tissue. Notably, when these mice expressed endothelial NOX5, there was a significant induction mainly in the expression of *Cav1* and *Glut4* (Figure 3, Figure 4 and Figure 5). Again, the in vitro model confirmed these results, and when 3T3-L1 adipocytes were treated with Glu + PA conditioned media coming from NOX5-expressing bEnd.3 endothelial cells, the expression of *Cav1* and *Glut4* was found to be significantly augmented (Figure 7).

Insulin-stimulated glucose uptake is accomplished through triggered translocation of the glucose transporter GLUT4 from intracellular vesicular reservoirs to the plasma membrane [44]. Membrane *caveolae*, where CAV1, IR-B, and GLUT4 colocalize, have been shown to be essential in this process. CAV1 activation by the IR upon insulin binding facilitates insulin signal transduction that leads to the translocation of GLUT4 to the plasma membrane [45,46,47]. Therefore, our results advocate that NOX5 expression in the endothelium helps to elicit an initial response against the excess of nutrients that contains a hyper-caloric diet such as the high-fat content diet used to feed the animals in this study. One effect of this early response is the enhanced insulin sensitivity of the white adipose tissue, which shows increased glucose uptake. The higher levels of *Cav1* and *Glut4* measured may form part of the necessary adjustments. Moreover, CAV1 is also involved with lipid accumulation. Studies performed with *Cav1* knockout mice have shown that a reduction of *Cav1* expression in the organism reflects in a higher hypertriglyceridemia [48,49]. As a result, the increase in *Cav1* expression would explain the lower weight gain and lower weights of adipose tissue from mice expressing NOX5 and fed with a HFD.

Finally, the fact that NOXs have been reported to be found within *caveolae* in endothelium [23,24], as well as in adipose tissue [19], and that they can interact with CAV1, raises the possibility of a cross-regulation, something that deserves further investigation. In regard to this, it is interesting the finding that ROS may enhance *CAV1* transcription in NIH-3T3-L1 fibroblasts [50].

**Figure 8:**
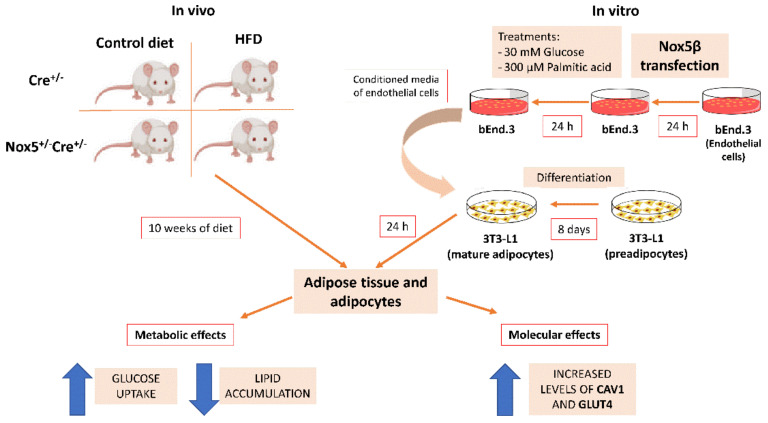
Graphical scheme of the experimental design and principal findings.

In summary, we found that the expression of NOX5 in the endothelium under obesity conditions is able to induce an upregulation of important insulin signalling intermediaries, such as *Ir*-*B*, *Cav1*, and *Glut4* in the neighbouring adipose cells. This is associated with an improved insulin-response increasing glucose uptake by the adipocytes and also in a reduced lipid accumulation (Figure 8). These results suggest that endothelial NOX5 may have a role in modulating a protective adipose tissue adaptation to the excess of nutrients caused by a high-fat diet in order to avoid, or at least delay, lipid accumulation and insulin resistance development.

## 4. Materials and Methods

### 4.1. Animals

A novel humanised knock-in mice for the endothelial expression of NOX5-B were employed in this study (Nox5+/−Cre+/−) [26]. Endothelial-specific CRE recombinase-expressing mice (CRE+/−) were used as control group (control CRE). Seven-week-old male mice were fed ad libitum with high-fat diet (HFD) or control diet for 10 weeks. HFD was a commercial obesogenic diet consisting of 60% calories as fat and 20% for both protein and carbohydrates (OpenSource Diet Product Data-D12492, Research Diets, New Brunswick, NJ, USA). At the time of sacrifice, tissue samples were rapidly extracted, weighted, and stored at −80 °C until they were processed. All in vivo experiments were performed in accordance with the European Communities Council Directives guidelines for the care and use of laboratory animals (2016/63/EU) and were approved by the University of Navarra Animal Research Review Committee (Protocol 106-17). Mice were randomly distributed in different cages with a maximum of 6 animals per cage.

### 4.2. Intraperitoneal Glucose Tolerance Test (IPGTT)

IPGTT was carried out at the ninth week of treatment, 1 week before sacrifice. First, mice were weighted and a 30% *w*/*v* glucose (Sigma Aldrich, St. Louis, MO, USA) solution was intraperitoneally administered at a concentration of 2 g/kg. Venous blood samples were obtained from the tail and glucose levels were determined using a glucometer (AccuCheck Aviva, Roche Diagnostics, Rotkreuz, Switzerland). Glucose measurements were recorded at 0, 20, 40, 60, 100, and 160 min after the administration.

### 4.3. Plasma Biochemistry Parameters

Blood was directly extracted from the heart immediately after mice were sacrificed. Blood samples were mixed with 0.1 M sodium citrate (Merck, Darmstadt, Germany) and maintained in ice for 15 min. Samples were then centrifuged (2000× *g* at 4 °C for 15 min) in order to obtain plasma that was stored at −80 °C.

Biochemistry parameters were determined from plasma samples in a Pentra C200 autoanalyzer (Horiba, Kyoto, Japan) with specific kits for each parameter.

### 4.4. Materials

Dulbecco’s modified Eagle’s medium (DMEM), foetal bovine serum (FBS), 0.25% *w*/*v* trypsin-EDTA, 1% *w*/*v* penicillin–streptomycin solution, phosphate-buffered saline (PBS), Opti-MEM, and Lipofectamine 3000 were purchased from Gibco (Thermo Fisher Scientific, Inc., Waltham, MA, USA). Insulin, water-soluble dexamethasone, 3-isobutyl-1-methylxanthine (IBMX), palmitic acid (PA), and glucose (Glu) were purchased from Sigma Aldrich (St. Louis, MO, USA). General chemicals reagents were purchased from Sigma Aldrich unless otherwise specified. Cell culture plastics were obtained from Corning (Thermo Fisher Scientific, Inc., Waltham, MA, USA).

### 4.5. Cell Culture

bEnd.3 (CRL-2299) and 3T3-L1 (CL-173) were obtained from ATCC (American Type Culture Collection, Manassas, VA, USA). Cells were grown in complete medium based on DMEM supplemented with 10% FBS and 0.1% *w*/*v* penicillin–streptomycin and were maintained in an incubator at 37 °C and 5% CO_2_. 3T3-L1 cells were differentiated into adipocytes. For that purpose, 1 × 10^6^ cells were seeded in 6-well plates, and 2 days after reaching confluence (day 0), they were incubated for 48 h in DMEM supplemented with 10% FBS, 1 µg/mL of insulin, 1 mM dexamethasone, and 0.5 mM IBMX. Cells were then incubated with DMEM containing 1 µg/mL of insulin for another 48 h. From days 4 to 8, cells were maintained with DMEM supplemented with 10% FBS. At day 8, cells were considered mature adipocytes. Differentiation process was confirmed by Oil Red staining as previously described [51].

In order to simulate obesity conditions, bEnd.3 cells, transfected or not, were treated with 30 mM glucose and 300 µM palmitic acid [52] for 24 h (Glu + PA conditioned media). After this time, the conditioned media was collected and added to mature adipocytes for 24 h.

### 4.6. Transient Transfection

The endothelial bEnd.3 cell line was transfected with the pcDNA3.2-NOX5-B expression plasmid [53] employing the Lipofectamine 3000 Transfection Kit according to manufacturer’s instructions. Briefly, 3 × 10^5^ cells per well were seeded in 6-well plates in a total volume of 2 mL. Then, 2.5 µg of plasmid were mixed with 5 µg of Lipofectamine 3000 in 250 µL of Opti-MEM. Transfection mix was added to the cells in the presence of DMEM without FBS and antibiotics for 5 h. Following this, complete medium was added, and incubation continued for 24 h. After this time, cells were treated for each assay. Control cells were transfected with an empty pcDNA3.2 vector.

### 4.7. Glucose Uptake Test

Glucose Uptake-GloTM Assay (Promega, Madison, WI, USA) was used for glucose uptake measurement. At day 8 of differentiation, 3T3-L1 cells were treated with conditioned media from bEnd.3 cells. After 24 h, glucose uptake test was performed.

For that purpose, treated adipocytes were incubated with 100 nM insulin for 10 min. Cells were then washed with PBS to eliminate all remnant glucose, and 1 mM 2-deoxyglucose (2DG) was added for 15 min. STOP solution (lysis buffer) and neutralization buffer were added for 10 min. Finally, labelled 2-deoxyglucose-6-phosphate (2DG6P) solution was added. Luminescence was read in a luminometer (Luminoskan Ascent, Thermo Fisher Scientific, Inc., Waltham, MA, USA) using an integration time of 0.3 s.

### 4.8. Quantitative Real-Time PCR

RNA from animal tissue samples (liver, mesenteric and epididymal fat) and 3T3-L1 adipocytes was extracted using Trizol (Thermo Fisher Scientific, Inc., Waltham, MA, USA) following standard protocols. cDNA was obtained from 2 µg of RNA using M-MLV Reverse Transcription enzyme (Thermo Fisher Scientific, Inc., Waltham, MA, USA). Gene expression was analysed using Taqman universal PCR Master Mix (Applied Biosystems, Cheshire, UK) and following a standard protocol: 50 °C for 2 min, 95 °C for 10 min, 40 cycles of denaturing at 95 °C during 15 s plus an annealing/extension step at 60 °C for 1 min. Specific Taqman probes (Applied Biosystems, Cheshire, UK) were used for each gene: insulin receptor β (Ir-B, Mm_01211875_m1), caveolin 1 (Cav1, Mm_00483057_m1), Slc2a4 (Glut4, Mm_00436615_m1), and glyceraldehyde 3-phosphate dehydrogenase(Gapdh, Mm_05724508_g1). All reactions were performed in triplicate. Gapdh was used as invariant internal control for qPCR and subsequent normalization. Relative quantification followed 2^−ΔΔCt^ method [54].

### 4.9. Western Blot

Proteins from tissue samples (liver, mesenteric and epididymal fat) and 3T3-L1 adipocytes were obtained using RIPA buffer (25 mM Tris-HCl, 150 mM NaCl, 0.1% *w*/*v* SDS, 1% *w*/*v* sodium deoxycholate, 1% *v*/*v* IGEPAL) supplemented with a protease inhibitor cocktail (Roche, Basel, Switzerland). A total of 30 µg of proteins were dissolved in loading buffer, and electrophoresis was performed at 120 V for 90 min in 10% SDS-PAGE. Proteins were then transferred onto a nitrocellulose membrane (GE Healthcare Amersham, Chicago, IL, USA) for 60 min at 0.35 A. Membranes were blocked with 5% *w*/*v* milk mixed with TBS (0.2 M Tris-HCl and 1.5 M NaCl at pH 7.4, 0.1% *w*/*v* Tween) for 60 min. Membranes were incubated overnight at 4 °C with specific primary antibodies at the appropriate dilutions: CAV1 (1:5000; Sc-894, Santa Cruz Biotechnology, Dallas, TX, USA), IR-B (1:1000; Sc-711, Santa Cruz Biotechnology), GLUT4 (1:1000; G4048, Sigma-Aldrich), Nitrotyrosine (1:500; AB5411, Merck; AB5411, Sigma-Aldrich), and B-ACTIN (1:10,000; A1978, Sigma-Aldrich). After 1 h incubation with secondary antibody at the appropriate dilutions, anti-rabbit (1:5000; NA934V, GE Healthcare) and anti-mouse (1:10,000; NA931V, GE Healthcare) membranes were developed by using Lumi-LightPlus Western Blotting Substrate (GE Healthcare Amersham, Chicago, IL, USA). Images were captured using a ChemiDOC XRS (Bio-Rad, Hercules, CA, USA) and analysed by Quantity One 1D software (Bio-Rad, Hercules, California, USA). B-ACTIN was used as invariant internal control for subsequent normalization.

### 4.10. Statistical Analysis

For groups following a normal distribution, results were expressed as mean ± standard error of the mean (SEM). For groups following a non-normal distribution, results were expressed as median with confidence interval. Normality and homogeneity of variance for each group were checked by Shapiro–Wilk and Flinger test, respectively. For in vivo data, comparisons among groups were performed employing two-way ANOVA (parametric) or Aligned Rank transformation ANOVA (non-parametric) test. For glucose curve t-test was used. For in vitro data, comparisons among groups were performed employing ANOVA (parametric) or Kruskal–Wallis (non-parametric) tests. Post-estimations were calculated using Holm correction. The statistical analysis was performed using RStudio (RStudio Team, 2020) and graphs were generated using GraphPad Prism 8 (GraphPad, San Diego, CA, USA).

## Data Availability

The data presented in this study are available on request from the corresponding author.

## Supplementary Materials

The following are available online at https://www.mdpi.com/1422-0067/22/5/2729/s1: Figure S1: Endothelial NOX5 expression increased albumin nitrotyrosine levels in mesenteric fat of mice fed a HFD for 10 weeks. Figure S2: Endothelial NOX5 expression tended to increase Cav1 levels in liver in mice fed with HFD for 10 weeks. Figure S3: NOX5 transfection of bEnd.3 cell line. Table S1: Biochemical parameters determined from plasma samples of mice.
