# Supplementary material for: Endothelial Nox5 Expression Modulates Glucose Uptake and Lipid Accumulation in Mice Fed a High-Fat Diet and 3T3-L1 Adipocytes Treated with Glucose and Palmitic Acid
Source: Int J Mol Sci. 2021 Mar 8;22(5):2729. doi: 10.3390/ijms22052729 (PMC7962974; doi:10.3390/ijms22052729)
Supplement: Supplementary file 1 [file ijms-22-02729-s001.zip › Supplementary material_JGarcia/Supplementary material.pdf]

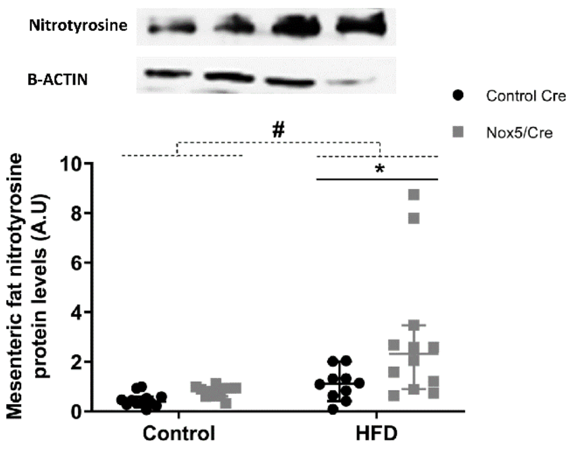

Figure S1. Endothelial NOX5 expression increased albumin nitrotyrosine levels in mesenteric fat of mice fed a HFD for 10 weeks. Representative Western blot and protein levels of albumin nitrotyrosine in total lysates of mesenteric fat. Control diet: Control Cre (n=12), Nox5/Cre (n=11); High-fat diet: Control Cre (n=10), Nox5/Cre (n=12). Values are expressed as median with confidence interval. Protein levels are relative to B-ACTIN. #p<0.05, diet differences (dotted lines); *p<0.05: genotype differences (solid lines). Statistical test used: Aligned Rank ANOVA.

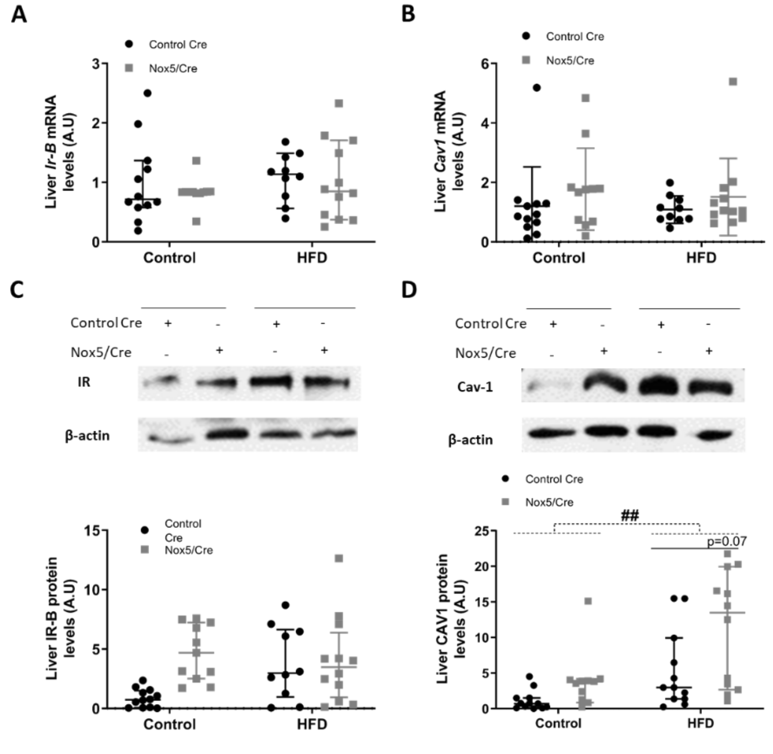

Figure S2. Endothelial NOX5 expression tended to increase *Cav1* levels in liver in mice fed with HFD for 10 weeks. (**A**) *Ir-B* mRNA levels in liver. (**B**) *Cav1* mRNA levels in liver. (**C**) Representative Western blot and protein levels of IR-B in total lysates of liver. (**D**) Representative Western blot and protein levels of CAV1 in total lysates of liver. Control diet: Control Cre (n=12), Nox5/Cre (n=11); High-fat diet: Control Cre (n=10), Nox5/Cre (n=12). Values are expressed as median with confidence interval. mRNA levels are relative to Gapdh. Protein levels are relative to B-ACTIN. ##p<0.01: diet differences (dotted lines); *p<0.05: genotype differences (solid lines). Statistical test used: Aligned Rank ANOVA.

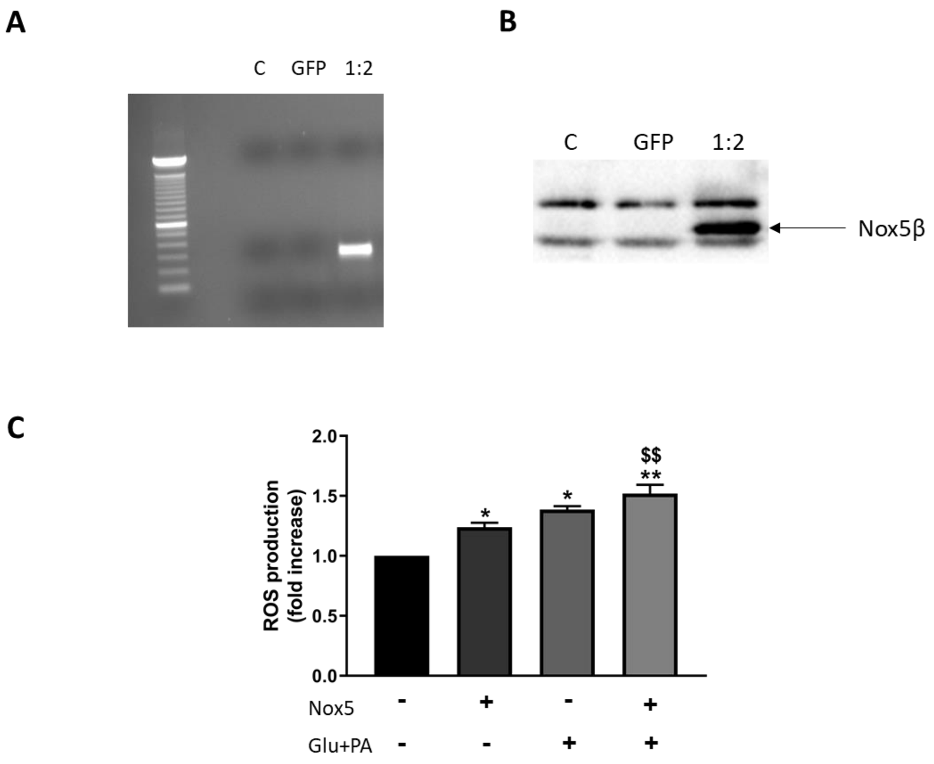

Figure S3. ***NOX5*** **transfection of bEnd.3 cell line.** (**A**) Conventional PCR for Nox5 detection after 24 hours of transfection. (**B**) Representative Western Blot of NOX5 after 24 hours of transfection. (**C**) Measure of NOX5 activity by Amplex Red after 24 hours of transfection. Cells were treated for 5 hours with 30 mM glucose and 300 μM palmitic acid to promote NOX5 activity. Amplex Red assay was performed following manufacturer instructions (#A12222, Thermo Fisher Scientific). Fold increase is relative to the control group (Nox(-)/Glu+PA(-)). Values are expressed as mean ± SEM. *p<0.05: differences relative to Nox(-)/Glu+PA(-); $$p<0.01: differences relative to Nox(-)/Glu+PA(+). Statistical test used: ANOVA.

Table S1. Biochemical parameters determined from plasma samples of mice. Concentration of cholesterol, HDL, TGC, Glucose and ALT was determined from plasma samples obtained before mice were sacrificed.

	Control		High-Fat	
	**WT**	**NC**	**WT**	**NC**
**Cholesterol (mg/dl)**	64,41±17,10	61,50±17,10	101,88±28,97^{xx}	98,58±13,43^{xx}
**HDL (mg/dl)**	34,42[4,84;42,70]	41,57[13,55;49,15]	49,47[34,72;55,91]	43,01[38,03;48,91]
**TGC (mg/dl)**	64,75±21,34	62,66±14,12	87±15,26^{x}	83,75±13,75^{x}
**Glucose (mg/dl)**	82,99±23,28	93,36±22,40	172,27±30,41^{xxx}	136,33±25,31$^{xxx;\#}$
**ALT (U/L)**	24,50[16,44;44,30]	25,05[22,20;27,40]	57,60[27,60;101,10]	37,70[21,10;41,55]
